# The Clavien–Dindo Classification for Body-Contouring Surgery Complications: Evaluation of 602 Cases

**DOI:** 10.3390/life14091120

**Published:** 2024-09-05

**Authors:** Michael S. Pollhammer, Dominik Duscher, Andrea Pagani, Maximilian Zaussinger, Raphael Wenny, Isabel Zucal, Manfred Schmidt, Lukas Prantl, Georg M. Huemer

**Affiliations:** 1 Section of Plastic, Aesthetic & Reconstructive Surgery, Kepler University Hospital Linz, Krankenhausstraße 9, 4020 Linz, Austria; ordination@drpollhammer.at (M.S.P.); maximilian.zaussinger@kepleruniklinikum.at (M.Z.); raphael.wenny@kepleruniklinikum.at (R.W.); manfred.schmidt@kepleruniklinikum.at (M.S.); office@drhuemer.com (G.M.H.); 2 Department of Plastic, Reconstructive & Aesthetic Surgery, University Hospital of Regensburg, Franz-Josef S. Allee 11, 93053 Regensburg, Germany; dominikduscher@me.com (D.D.); lukas.prantl@ukr.de (L.P.); 3 Doctoral Degree Program in Medial Science (Ph. D.), Paracelsus Medical University, Strubergasse 21, 5020 Salzburg, Austria; 4 Department of Plastic, Reconstructive and Aesthetic Surgery, Ente Ospedaliero Cantonale, 6900 Lugano, Switzerland; isabel.zucal@icloud.com

**Keywords:** Clavien–Dindo classification, postoperative complications, body contouring, reconstructive surgery

## Abstract

**Background**: Due to the high frequency of postoperative complications after body-contouring surgeries, the need for a unifying postoperative complication grading system that correlates with outcomes is of key importance. Here we therefore consider the application of the Clavien–Dindo classification to evaluate postoperative complications after body-contouring surgeries. **Methods**: A retrospective study on 602 patients who underwent body-contouring surgery between 2009 and 2015 at our institution was performed. The length of hospital stays, age, sex, follow-up visits, and postoperative complications were evaluated and classified using the Clavien–Dindo classification. **Results**: We raised a total of 672 body-contouring procedures on 602 patients (563 female, 39 male). According to the Clavien–Dindo System, the severity of postoperative complications following body-contouring procedures was significantly correlated with the duration of hospitalization (mean 5.8 ± 2.7 days) and the number of follow-up visits (mean 4.4 ± 4.7). **Conclusions**: The Clavien–Dindo classification offers a valid prediction for postoperative hospital stay and the number of follow-up visits after body-reshaping surgery. By becoming a validated and reliable grading system that correlates patients’ outcomes after body-contouring procedures, this classification has the potential to significantly improve patients’ healthcare and quality of life.

## 1. Introduction

The rapid increase in bariatric surgeries has led to a significant rise in body-reshaping procedures [1,2,3]. Despite numerous advancements, the complication rate following body-contouring surgeries remains substantially higher compared to other procedures [4]. A definitive consensus on classifying the severity of postoperative complications in relation to surgical and clinical outcomes is still lacking. Descriptive and quantitative terms such as “minor”, “major”, or “life-threatening” complications have been used non-specifically among authors, hampering the proper interpretation of outcome data across different procedures, centers, and surgeons [5]. Consequently, there is an urgent need for a widely accepted grading system for postoperative complications after body-contouring surgery that correlates with outcomes. This would work to compare the risks of various body-contouring procedures and provide an adequate, standardized quality assessment for therapies in this relatively new patient population.

In 1992, Clavien et al. [6] proposed a valuable classification model for postoperative complications in general surgery. This model used the treatment method required to address a complication as the basis for grading the complication itself in an objective, standardized, and reproducible manner. Twelve years later, Dindo et al. [7] modified this classification based on their experience and validation in a cohort of 6336 patients. Like the original classification, the treatment option used to manage an adverse event or reaction remained the cornerstone for grading the severity of postoperative complications. The updated Clavien–Dindo classification system provides a validated and reliable means of correlating surgical and clinical outcomes [8,9]. Over the last decade, the Clavien–Dindo classification has gained popularity among surgeons and has been tailored for numerous surgical specialties such as General Surgery [9], Neurosurgery [10,11], Pediatric Surgery [12], Otolaryngology [13,14], Urology [15], Orthopedics [16], Oncology [17], Gynecology [18], and Plastic and Reconstructive Surgery [19] (Table 1).

At present, monitoring postsurgical events is one of the most reliable hallmarks of outcome and quality measurements. Strong et al. [20] widely described how the Memorial Sloan Kettering Cancer Center’s Surgical Secondary Events (SSE) System© enables hospitals to capture information about oncology-related surgical complications in a consistent and reproducible way. The data that are captured via the SSE System are available to review postoperative morbidity and mortality for the comparison of institutions for medical-intervention decision-support projects and the final promotion of quality improvement in patient management [21]. Katayama et al. [22] developed the Japan Clinical Oncology Group postoperative complications (JCOG PC)-Criteria, an extended complication grading system that standardizes the terms used in the traditional Clavien–Dindo classification and provides more detailed grading guidelines. After putting together members from nine surgical study groups, the authors could achieve, via the JCOG PC Criteria, a more precise comparison of the frequency of postoperative complications among trials across many different surgical fields. Slankamenac et al. [23] proposed, in 2013, the Comprehensive Complication Index (CCI), a continuous scale ranging from 0 to 100 that calculates the sum of all complications weighted for their severity. Because of this increasingly high interest in complication grading systems, we considered the potential applicability of the Clavien–Dindo Classification on body-reshaping procedures. Postoperative complications not only impact the patient’s immediate postoperative recovery but can also influence long-term outcomes and the overall success of the surgery. A standardized classification system like the Clavien–Dindo model could be essential for accurately documenting these complications, comparing outcomes across different studies, and most importantly, improving patient healthcare and quality of life [24,25].

The possibility of applying a classification system that is already established in other surgical sectors to our field of interest is a particularly interesting point of view that could lead to a significant improvement in our health service and a qualitative improvement of the patient’s health and life quality. The application of the Clavien–Dindo classification in body-contouring surgery has not been validated so far. Therefore, here we present our preliminary study to explore the feasibility of using the Clavien–Dindo classification in body-reshaping procedures.

## 2. Methods

We collected all the patients who underwent body-contouring surgery at our University Hospital in Linz (Austria) between June 2009 and January 2015. Written informed consent was collected from all patients and the study was carried out in accordance with the Declaration of Helsinki. Different body-contouring procedures including lower and upper body lifts, abdominoplasty, breast reduction, mastopexy, brachioplasty, and thigh lift were included (Table 2). Data such as sex, age, length of hospital stays, follow-up visits after surgery, and postoperative complications were considered. Postoperative complications were reviewed and classified by the first author and re-reviewed by the senior author to confirm the initial classification. In order to validate the Clavien–Dindo classification for complication severity in body-contouring surgery, we compared various grades of complications with the length of hospital stay and the number of follow-up visits during the postoperative period.

### 2.1. Classification of Postoperative Complications

All complications that occurred in our patient population were assessed based on the Clavien–Dindo classification (Table 1). If more than one complication occurred, we considered the most severe complication to determine the right complication grade.

### 2.2. Statistical Analysis

Statistical analysis was performed using SPSS^TM^ (Version 22.0, IBM, New York, NY, USA) and R statistical software (R Foundation for Statistical Computing. Vienna, Austria. www.R-project.org). Possible correlations between Clavien–Dindo complication grades and the length of hospital stay as well as the number of follow-up visits were analyzed using the bivariate Spearman rank correlation test. For all statistical analyses, the level of significance was considered *p* < 0.05.

## 3. Results

### 3.1. Patients Data

In total, 602 patients were recruited between June 2009 and January 2015 after body-contouring surgery at our institution. The study population included 563 females and 39 male patients (average age 40 ± 11.3 years). In the mentioned time period, a total of 672 body-contouring procedures were performed, including 98 body lifts, 180 abdominoplasties, 214 breast reductions, 72 mastopexies, 71 thigh lifts, and 37 brachioplasties. In 64 cases, 2 or more procedures were performed simultaneously. The mean length of hospital stay was 5.8 ± 2.7 days and the mean number of follow-up visits during the postoperative period was 4.4 ± 4.7, including patients with and without postoperative complications (Table 3).

### 3.2. Complication Data and Validation

In 274 of 602 patients (41.0%), 1 or more complications were identified. Minor complications like wound-healing deficits, which are typically encountered in body-contouring surgery, were recorded in 18.1% of the patients and classified as grade 1. Grade 2 complications with the need for pharmacological treatment were found in 8.9% of patients. The necessity of surgical intervention was divided into subgroups, analog to the modified Clavien–Dindo classification into grades 3a and 3b, depending on the necessity of general anesthesia. In our patient population, grade 3a complications included seromas and hematomas, which are easily treated by needle puncturing. These occurred in 15.6% of our patients. Grade 3b complications required surgical revision with general anesthesia. Grade 3b complications were noted in only 2.8% of all cases. No complications classified higher than Grade 3b were recorded in our study (Table 4).

**Figure 1:**
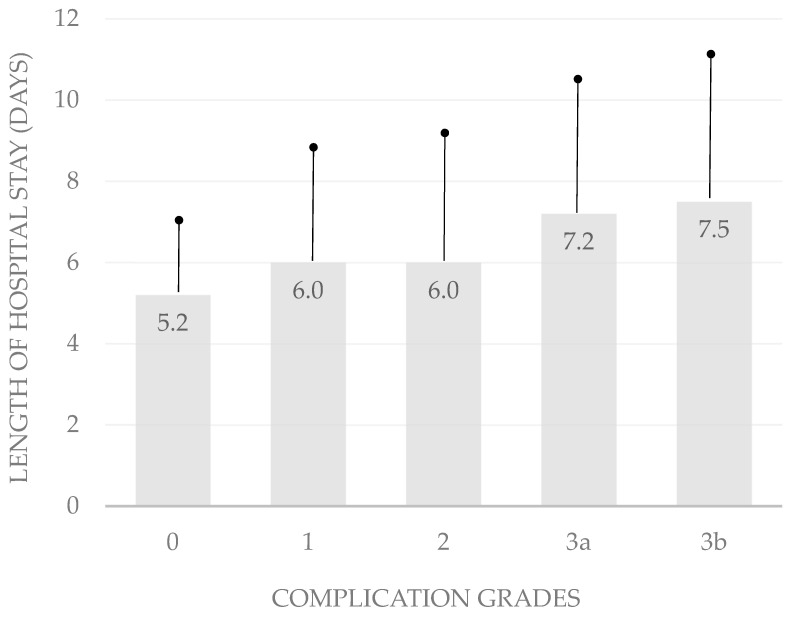
**Hospital stay by complication severity**. This bar chart illustrates the significant correlation between complication severity and the mean length of hospital stay, highlighting the increasing duration with higher complication grades.

It is difficult to rank abdominoplasties, breast reductions, mastopexies, thigh lifts, and brachioplasties by complexity. Because the grading of complexity in body-contouring surgery is challenging, we chose the length of hospital stay and the number of follow-up visits as indicators for the impact of complications on surgical outcomes and the validation of the Clavien–Dindo classification (Table 5). The classification correlated significantly with the length of hospital stay (*p* < 0.05; bivariate Spearman rank correlation test). In patients without complications, the mean duration of hospitalization was 5.2 ± 2.2 days. The average length of hospital stay was 6.0 ± 2.7 days in patients with complications classified as grade 1, 6.0 ± 3.1 days in those with grade 2 complications, 7.2 ± 3.2 days in those with grade 3a complications, and 7.5 ± 3.6 days in patients with complications rated 3b (Table 5; Figure 1).

**Figure 2:**
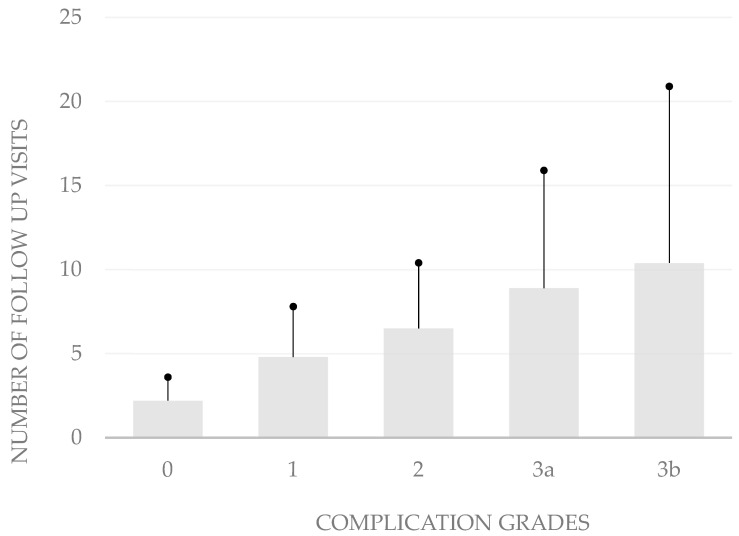
**Number of follow-up visits by complication severity**. In aggregate, these data show that the modified Clavien–Dindo classification is well suited to depict the severity of complications after body-contouring surgery.

The grading of the complications also correlates with the number of follow-up visits after hospitalization (*p* < 0.05, bivariate Spearman rank correlation test). The average number of follow-up consultations was 2.2 ± 1.4 in patients without complications. When patients developed grade 1 complications, the mean number of follow-up visits was only 4.8 ± 3.0, while it was 6.5 ± 3.9 in those who developed grade 2 complications, 8.9 ± 7.0 in grade 3a complications, and 10.4 ± 10.5 in grade 3b complications (Figure 2).

## 4. Analysis and Discussion

The prevalence of obesity is increasing steadily and, as a result, body-contouring surgery in patients after massive weight loss is one of the fastest growing fields in plastic surgery [3]. More than 20 years ago, Kolotkin et al. [26] showed how the dramatic excess of overstretched skin following massive weight loss often negatively affects the quality of patient’s lives and causes psychological and even physical issues. In these cases, body-reshaping procedures offer patients the possibility of completing their body transformation after weight loss and achieving a satisfying appearance [27]. Although body-contouring procedures are considered to be safe elective procedures, the risk for postoperative complications in body-reshaping surgeries seems to be higher than in other fields of plastic surgery.

According to the current literature, 85% of massive weight loss patients seek body-contouring surgeries with a calculated number of 46,577 procedures in 2020 [24]. Because of their intrinsic clinical complexity, massive weight loss patients present insidious post-operative complications such as seroma, wound infection, dehiscence, necrosis, lymphorrhea, asymmetry, and thrombosis [28,29,30]. A systematic review and meta-analysis of Marouf et al. [31], including 25 studies of a total of 561 articles, reported an overall weighted rate of postbariatric body-contouring surgical complications of 31.5%. Seroma was, with a weighted rate of 12.7–13.9%, the most frequent complication. Furthermore, regarding risk factors, the analysis indicated that a Body Mass Index (BMI) of less than 30 kg/m^2^ and a low mean weight of resected tissue were associated with fewer complications. In a cohort study including 153 individuals who underwent a total of 198 body-contouring surgeries after massive weight loss, Garcia Botero et al. [32] reported an overall complication rate of 55.5% with a major complication rate of 13% and a minor complication rate of 87%. Even a recent study from part of our group showed, in 788 body-contouring procedures, a total number of 362 (46%) complications, 152 (19%) of which were described as major and 210 (27%) as minor complications (i.e., delayed healing, unfavorable scarring, hematoma, and seroma). Thighplasty showed the highest complication rate in our series (63%) and represents one of the most challenging surgery in this field [24]. Despite several hypotheses, such as mechanical stress of the soft tissues before bariatric surgery and a problematic nutritional state, an adequate explanation for the high complication rate in this population is still lacking.

Some of the most relevant studies regarding this challenge were written recently by Bertheuil et al., Losco et al., and Gusenoff et al. [33,34,35]. Bertheuil et al. [33] examined the risk factors associated with complications following medial thighplasty, identifying preoperative BMI as a significant predictor of postoperative issues. Their findings highlight the complexity of selecting appropriate candidates for surgery and the critical need for well-defined guidelines to optimize patient outcomes. On the other hand, Gusenoff et al. [34] provided a detailed analysis of the complication profiles for medial thighplasty in the massive weight loss population, reporting a high incidence of minor wound-healing problems, especially in cases involving full-length vertical thighplasty. Their results suggest that factors such as hypertension, age, and concurrent liposuction may increase the risk of complications, underscoring the importance of comprehensive preoperative assessments and thorough patient counseling. Finally, Losco et al. [35] proposed a novel “helix thigh lift” technique to address severe gynoid deformities commonly seen after massive weight loss. This approach, which combines vertical and horizontal pulling axes, has resulted in high levels of patient satisfaction and a manageable complication rate. The study emphasizes the necessity of individualized treatment plans, while also identifying age as a factor significantly associated with the development of complications. If, on one hand, these studies contribute to a deeper understanding of the complexities involved in thigh contouring after massive weight loss, on the other hand, they underscore the need for tailored surgical approaches to minimize complications and enhance patient outcomes.

At present, the rising costs of the healthcare system are becoming a worldwide concern, and complications following surgery are known to be a major factor in increasing hospital expenses. By analyzing 38 studies on pancreatic, urologic, gynecological, thoracic, and hepatic surgery, a systematic review by Patel and colleagues [36] showed that complications lead to higher resource use and hospital costs compared with surgical procedures without complications. Specifically, Poyatos et al. [37] reported that a single complication in post-bariatric surgery increases the average hospital cost per patient by 2.96. As a consequence, quality assessment has gained increasing attention among surgeons treating massive weight loss patients. Our article shows that, even for body-contouring procedures, the Clavien–Dindo classification can offer an easy and reliable rating system for postsurgical complications by focusing on their therapeutic consequences.

Upon analyzing our results, we observed significant variability within the classification groups concerning hospital length of stay and follow-up visits. For instance, a complication classified as grade 3, which requires surgical intervention, may be resolved quickly and result in a rapid discharge. Conversely, a minor complication that does not necessitate pharmacological or surgical intervention may still require extended observation, leading to a prolonged hospital stay or an increased number of follow-up visits. However, when considering the average hospital stay and follow-up visits, we found that more severe complications generally correlated with longer hospital stays and more follow-up visits. This high variability suggests that the classification system could be refined to better predict the consequences of complications. Nevertheless, we identified a strong correlation between the Clavien–Dindo classification and the duration of hospitalization, as well as a clear link between higher classification grades and the number of follow-up visits. Notably, no grade 4 or higher complications were observed in our study. Although the Clavien–Dindo classification effectively captures the spectrum of postoperative complications, it does not account for aesthetic outcomes and patient satisfaction, which are critical in plastic, reconstructive, and aesthetic surgery. This limitation highlights an area for potential improvement in the classification system.

Despite the accuracy with which the data were collected, the study has some limitations. First, its retrospective design limits the ability to establish a direct causal relationship between the identified risk factors and the complications observed after body-contouring surgeries. The reliance on existing medical records may also lead to incomplete data collection, as some variables or patient details could have been underreported or missing from the documentation. Second, while the study uses the Clavien–Dindo classification to effectively categorize postoperative complications, it does not account for aesthetic outcomes or patient satisfaction, which are critical parameters in body-contouring surgeries. This omission partially restricts an evaluation of surgical success, as the classification primarily focuses on clinical complications rather than the overall quality of the surgical results from the patient’s perspective.

The application of the Clavien–Dindo classification in body-contouring surgery has not been validated previously. Thus, the aim of this preliminary study is to explore the feasibility of using the Clavien–Dindo classification in body-contouring surgery to provide a standardized interpretation of surgical and clinical outcomes and ultimately improve the quality of care for this patient population. By standardizing the classification of complications, clinicians can better assess the risks and benefits of various body-contouring procedures, identify areas for improvement, and develop targeted interventions to enhance patient outcomes. In conclusion, the Clavien–Dindo classification, with its objective and reproducible approach, offers a promising framework for this purpose. Further validation in the context of body-contouring procedures is essential to ensure its effectiveness in this unique patient population. By integrating clinical and cellular perspectives, healthcare providers can enhance postoperative quality of life and achieve better surgical outcomes for patients undergoing body-contouring procedures after massive weight loss.

## 5. Conclusions

This study demonstrates that the Clavien–Dindo classification provides a reliable and standardized method for categorizing postoperative complications in body-contouring surgery, particularly for patients who have undergone massive weight loss. Our findings indicate a significant correlation between the severity of complications, as classified by the Clavien–Dindo system, and both the length of hospital stay and the number of follow-up visits. This suggests that the Clavien–Dindo classification can serve as a useful tool for predicting and managing postoperative outcomes in this patient population. However, the study also highlights certain limitations of the Clavien–Dindo classification, particularly its lack of consideration for aesthetic outcomes and patient satisfaction, which are critical in plastic and reconstructive surgery. Further research is needed to refine the classification system to better predict the impact of complications on these factors. Overall, the application of the Clavien–Dindo classification in body-contouring surgery after massive weight loss offers a promising framework for standardizing the assessment of surgical outcomes. By adopting this classification, clinicians can improve the quality of care, enhance patient safety, and develop more effective, individualized treatment plans. Future validation studies are essential to confirm its utility and adaptability in this unique surgical context.

## Figures and Tables

**Table 1:** **The Clavien–Dindo Classification**. The Clavien–Dindo classification is a widely used system for categorizing surgical complications based on their severity. It ranges from Grade I, which involves minor deviations from the normal postoperative course, to Grade V, which denotes patient death. Grades II and III involve complications requiring pharmacological treatment or surgical intervention, while Grade IV includes life-threatening complications requiring intensive care.

Classification of Surgical Complications (Clavien-Dindo, 2004)	
Grade	Definition
**I**	Any deviation from the normal postoperative course without the need for pharmacological treatment or surgical, endoscopic, and radiological interventions. Allowed therapeutic regimens are drugs (antiemetics, antipyretics, analgetics, diuretics, electrolytes) and physiotherapy. This grade also includes wound infections opened at the bedside
**II**	Requiring pharmacological treatment with drugs or other than such allowed for grade I complications; Blood transfusions and total parenteral nutrition are also included
**III**	Requiring surgical, endoscopic or radiological intervention
**IIIa**	Intervention not under general anesthesia
**IIIb**	Intervention under general anesthesia
**IV**	Life-threatening complications (including CNS complications) * requiring IC/ICU management
**IVa**	Single organ dysfunction (including dialysis)
**IVb**	Multiorgan dysfunction
**V**	Death of a patient
Suffix “**d**”	If the patient suffers from a complication at the time of discharge, the suffix “d” (disability) is added to the respective grade of complication. The label indicates the need for a follow-up.

* Brain hemorrhage, ischemic stroke, subarrachnoidal bleeding, but excluding transient ischemic attacks. CNS, central nervous system; IC, intermediate care; ICU, intensive care unit.

**Table 2:** **Body-contouring procedures between 2009 and 2015**. The table shows the type of surgeries performed at our institution that were included in our study (left side) and the different variables that were assessed (right side).

**Body-Contouring Procedures (2009–2015)**	**Sex** **Age** **Length of hospital stays** **Follow up visits** **Postoperative complications**
Upper and Lower Bodylifts	
Abdominoplasty	
Breast Reduction Surgery	
Mastopexy	
Brachioplasty	
Thigh Lift	

**Table 3:** **Patients and numbers of procedures between 2009 and 2015**. The table shows the number of surgeries performed at our institution that were included in our study (left side) and the variables that were assessed (right side). In 64 cases, 2 or more procedures were performed simultaneously.

**Year 2009–2015** **602 Patients [563F–39M]—Age 40 ± 11.3 Years** **672 Body-Contouring Procedures**	**Sex** **Age** **Length of hospital stays** **Follow up visits after surgery** **Postoperative complications**
98 Upper and Lower Bodylifts	
180 Abdominoplasties	
214 Breast Reduction Surgeries	
72 Mastopexies	
37 Brachioplasties	
71 Thigh Lifts	
In 64 cases two or more procedures were performed simultaneously	

**Table 4:** **Complication data and validation.** In 274 out of 602 patients (41.0%), 1 or more complications were identified. Minor complications occurred in 18.1% and were classified as grade 1. Grade 2 complications requiring pharmacological treatment were found in 8.9% of patients. Grade 3a complications, such as seromas and hematomas treated without general anesthesia, occurred in 15.6%, while grade 3b complications, requiring surgical revision with general anesthesia, were noted in 2.8% of cases. No complications higher than grade 3b were recorded.

**One or More Complications (274 of 602 Patients)**				No Complications (328 of 602 Patients)
**41.0%**				59.0%
Minor Complications 18.1%	Pharmacological treatment 8.9%	Surgery under GA 15.6%	Surgery not under GA 2.8%	
I	II	III A	III B	

**Table 5:** **Correlation between complication severity, length of hospital stay, and number of follow-up visits**. The table summarizes the correlation between the severity of complications, the length of hospital stay, and the number of follow-up visits. The data are presented as mean duration in days (±standard deviation) for each complication grade.

Postoperative Complications	Hospital Stay (Days)	Follow up Visits (N°)
No	5.2 ± 2.2	2.2 ± 1.4
Grade I	6.0 ± 2.7	4.8 ± 3.0
Grade II	6.0 ± 3.1	6.5 ± 3.9
Grade IIIa	7.2 ± 3.2	8.9 ± 7.0
Grade IIIb	7.5 ± 3.6	10.4 ± 10.5

## Data Availability

Data is contained within the article.
